# Determinants of physical activity and exercise in healthy older adults: A systematic review

**DOI:** 10.1186/1479-5868-8-142

**Published:** 2011-12-28

**Authors:** Margot A Koeneman, Marieke W Verheijden, Mai J M Chinapaw, Marijke Hopman-Rock

**Affiliations:** 1 Body@Work, Research Center for Physical Activity, Work and Health, TNO-VU University Medical Center, Amsterdam, The Netherlands; 2 Department of Public and Occupational Health, EMGO+ Institute for Health and Care Research, VU University Medical Center, Amsterdam, The Netherlands; 3 TNO, Leiden, The Netherlands

**Keywords:** Prevention, Behaviour change, Determinants, Active lifestyle, Aged

## Abstract

**Background:**

The health benefits of regular physical activity and exercise have been widely acknowledged. Unfortunately, a decline in physical activity is observed in older adults. Knowledge of the determinants of physical activity (unstructured activity incorporated in daily life) and exercise (structured, planned and repetitive activities) is needed to effectively promote an active lifestyle. Our aim was to systematically review determinants of physical activity and exercise participation among healthy older adults, considering the methodological quality of the included studies.

**Methods:**

Literature searches were conducted in PubMed/Medline and PsycINFO/OVID for peer reviewed manuscripts published in English from 1990 onwards. We included manuscripts that met the following criteria: 1) population: community dwelling healthy older adults, aged 55 and over; 2) reporting determinants of physical activity or exercise. The outcome measure was qualified as physical activity, exercise, or combination of the two, measured objectively or using self-report. The methodological quality of the selected studies was examined and a best evidence synthesis was applied to assess the association of the determinants with physical activity or exercise.

**Results:**

Thirty-four manuscripts reporting on 30 studies met the inclusion criteria, of which two were of high methodological quality. Physical activity was reported in four manuscripts, exercise was reported in sixteen and a combination of the two was reported in fourteen manuscripts. Three manuscripts used objective measures, twenty-two manuscripts used self-report measures and nine manuscripts combined a self-report measure with an objective measure. Due to lack of high quality studies and often only one manuscript reporting on a particular determinant, we concluded "insufficient evidence" for most associations between determinants and physical activity or exercise.

**Conclusions:**

Because physical activity was reported in four manuscripts only, the determinants of physical activity particularly need further study. Recommendations for future research include the use of objective measures of physical activity or exercise as well as valid and reliable measures of determinants.

## Background

As the proportion of older adults in Western societies continues to grow, so does their average life expectancy. Even though regular physical activity (PA: unstructured activities incorporated in daily life) and participation in exercise (EX: structured and planned activities) have many health benefits, levels of PA and EX decrease with increasing age [1]. The age related decline in physical capacity is experienced as an increased effort needed to perform daily activities, which could ultimately lead to avoidance of PA and EX [2]. This is a worrisome trend that can be prevented by adopting or maintaining regular PA and EX into old age [2,3]. Even when optimal levels of activity cannot be achieved, increasing PA and EX participation can still induce health benefits [1]. The limited success in getting and keeping older adults physically active [4,5] shows a great need for knowledge of determinants of PA and EX.

Recent literature reviews on determinants of PA and EX in older adults have included Randomized Controlled Trials (RCTs) and prospective studies to draw conclusions on causal relationships [6,7]. Martin and Sinden [6] explored predictors of older adults' adherence to exercise interventions and suggested that different variables predict adherence at different time points, also depending on the type of exercise (e.g. aerobic or strength programs). Adherence to exercise was best predicted by health and health indicators, such as being fit, non-smoking, having an active and healthy lifestyle and psychological determinants, such as high self-efficacy. Van Stralen and colleagues [7] explored determinants of initiation and maintenance of physical activity among older adults and suggested that determinants are partly phase-specific and differ for initiating (up to six months) or maintaining (more than six months) physical activity.

It remains, however, unclear how determinants may differ between PA and EX. Considering the divers nature of physical activity and exercise together with the finding of Martin and Sinden [6] that different types of exercise were predicted by different determinants, it seems plausible that determinants of physical activity and exercise are not the same. We intended to build upon previous work [6,7]. In addition, we wanted to differentiate between determinants of PA and EX in healthy older adults only. Therefore, our aim was to assess the determinants of PA as well as EX in healthy older adults, considering the methodological quality of the included studies.

## Methods

### Literature search

We conducted a literature search in the databases PubMed/Medline and PsycINFO/OVID for peer reviewed manuscripts published in English from 1990 onwards. We started the literature searches in May 2009, and conducted updates until January 2011. Search terms included physical activity, exercise and terms describing the target population (e.g., older adults, aged and aging). The full search strategy can be obtained from the corresponding author. Additional searches were performed using reference lists of review articles retrieved through the original search.

### Manuscript selection

Two researchers (MK and MV) independently performed an initial selection for eligibility based on the titles of the manuscripts. Three of the authors (MK, MH and MC) independently assessed the remaining abstracts and included manuscripts that met the following criteria: 1) population: community dwelling healthy older adults, aged 55 and over (based on mean age and/or age range); and 2) prospective manuscripts reporting determinants of PA or EX. International guidelines [3] do not provide a clear cut point for "old age". We therefore arbitrarily chose to include studies with a population aged 55 and older. Manuscripts reporting on a specific subsample of the older population (e.g. ethnicity, IQ, confined geographic area or diagnosed diseases) were excluded. Similarly, manuscripts reporting on interventions to prevent falls or obesity were excluded.

### Categorisation

Manuscripts were categorised based on: outcome measure (PA or EX, and objective or self-report). We used the definition by Caspersen et al. [8] to categorise the outcome measure as either PA or EX. PA was defined as 'occupational physical activity, household activities and walking/strolling for entertainment, social goals or transport'. EX was defined as 'physical activity, which is planned, structured, and repetitive, with the specific goal to maintain or improve physical fitness'. In line with this categorisation, walking can either be categorised as PA, when participants walk as a form of transport, or as EX, when participants are instructed to have planned, repetitive walks of specific duration. The outcome measure was further categorised as either objective (such as: pedometers and accelerometers) or self-report (such as: exercise logs, questionnaires and recording of class attendance).

### Methodological quality assessment and evidence synthesis

The methodological quality of the selected studies was assessed based on the items on validity and precision derived from a checklist from Chinapaw et al., Brown et al. and Uijtdewilligen et al. [9-11]. The checklist is presented in Table 1 and consists of 8 items describing four categories (study population and participation; study attrition; data collection; and data analyses). Two authors (MK, MV) and a trained statistician independently appraised the methodological quality of the included studies. Items were rated "+" if the requested information was present in the paper and criteria were met. Items were rated '-' if the requested information was present in the paper and criteria were not met. Items were scored "?" if insufficient information was provided. If one of the selected manuscripts referred to another publication for information on the checklist items, this publication was retrieved and the information was extracted. Disagreement was resolved through discussion. The overall methodological quality of a study is expressed as a percentage calculated from the total number of "+" scores divided by 8. Manuscripts with a percentage of 70% or more were considered high quality [11].

**Table 1:** Criteria list for assessment of the methodological quality of prospective studies

Criteria:	(rating of criteria: '+' = yes, '-' = no, '?' = not or insufficiently described)
**Study population and participation (baseline): The study sample represents the population of interest on key characteristics**	1. Participation rate at baseline at least 80%, or if the non-response was not selective (show that baseline study sample does not significantly differ from population of eligible subjects)

**Study attrition: Loss to follow-up is not associated with key characteristics (i.e. the study data adequately represent the sample)**	2. Response at short-term follow-up (up to 12 months) was at least 80% of the number of participants at baseline and response at long-term follow-up was at least 70% of the number of participants at baseline 3. Not selective non-response during follow-up measurement(s) a '+' is given only if non-selective dropout on key characteristics (age, gender, relevant determinants, and outcome measure) is reported in the text or tables

**Data collection:**	4. Adequate measurement of PA, EX or PA/EX: objective measurement and not by self-report (self-report = '-', no/insufficient information = '?') 5. Determinants of PA or EX behaviour were measured with a reliable tool '+' is given only if measures of determinants showed ICC/KAPPA of ≥0.60 (or Pearson correlation above .70) assessed within the appropriate target population. For biological variables, a '+' was given only if a standardised protocol was followed, and trained researchers assessed the determinants. A '+' was also given for age, gender, ethnicity, marital status, socio-economic status, employment status, education, income, intervention condition and objective assessment of environmental characteristics (proximity of parks and weather conditions). Expressed as the number of cases in which the measurement of determinants were scored +, divided by the total number of determinants measured in all studies. 6. Determinants of PA or EX behaviour were measured with a valid tool '+' is given only if measures of determinants showed correlations of ≥0.40 with other similar constructs measured within the appropriate target population. For PA, EX or PA/EX variables (e.g. past physical activity), a '+' was given only if variables were assessed with an objective measurement instrument (e.g. accelerometer/pedometer). For biological variables, a '+' was given only if a standardised protocol was followed, and trained researchers assessed the determinants. A '+' was also given for age, gender, ethnicity, marital status, socio-economic status, employment status, education, income, intervention condition and objective assessment of environmental characteristics (proximity of parks and weather conditions). Expressed as the number of cases in which the measurement of determinants were scored +, divided by the total number of determinants measured in all studies.

**Data analyses:**	7. The statistical model used was appropriate 8. The number of cases was at least 10 times the number of the independent variables

Because of the heterogeneity of the methods used in the included manuscripts statistical pooling of data was not possible. Instead, we applied a best evidence synthesis similar to Chinapaw et al. and Uijtdewilligen et al. [9,11]. This synthesis takes the methodological quality, the number of studies and the consistency of the evidence into account.

The evidence for the relationship between determinants and behavioural outcome (PA, EX or PA/EX) was rated *strong *(consistent findings in multiple (≥2) high methodological quality studies), *moderate *(consistent findings in one high quality and at least one low methodological quality studies or consistent findings in multiple (≥2) low methodological quality studies) or as *insufficient *(only one study available or inconsistent findings in multiple (≥2) studies). Results were considered to be consistent when at least 75% of the studies showed significant results in the same direction. When at least 75% of the studies found no significant association this would be considered insufficient evidence for an association ("no evidence for an association").

### Data extraction

**Figure 1:**
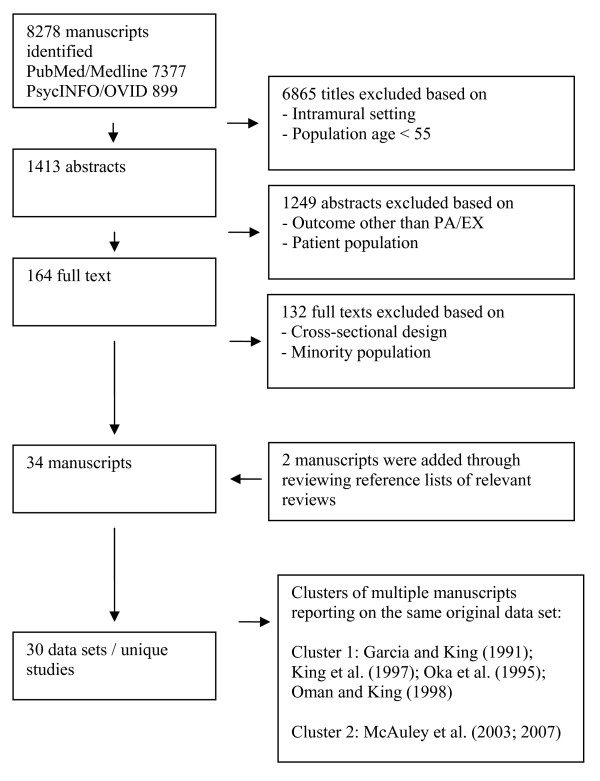
Flowchart of study selection process

For each individual manuscript, determinants were categorized as '+' (having a significant positive association with PA/EX), '-' (having a significant negative association with PA/EX), or '0' (having no association with PA/EX). Significance levels were set at p < 0.05. Notably, when manuscripts were reviewed, it appeared that multiple manuscripts reported on the same original data set. These clusters of manuscripts were made identifiable in Figure 1 and Table 2 and 3. If separate manuscripts reporting on the same study described similar associations, they represent a single count in the evidence synthesis. If separate manuscripts reporting on the same study described different associations, they each represent a count in the evidence synthesis.

**Table 2:** Manuscripts reporting on determinants of physical activity and exercise in healthy older adults

Reference		**Design: **Intervention study, observational study or randomized controlled trial **Age: **Mean (SD) or [Range]left **Quality score: **%	**Intervention: **Content **Outcome measure: **Self-report or objective + instrument **Outcome: **unstructured physical activity **(**PA), structured exercise (EX) or a combination of physical activity and exercise (PA/EX) - for definitions see main text	**Determinants: **Positive association (+), negative association (-) or no association (NS)
[24] Boyette et al. 1997 U.S.		**Design:** Intervention study **Age: **71.3 (4.6) **Quality score: **29	**Intervention:** Strength training intervention with considerable support from staff and program organization aimed at convenience **Outcome measure:** Self-report PEP **Outcome:** "Do you exercise for muscular strength and endurance?"/"Do you exercise for flexibility? (EX)	Satisfaction with exercise routine (+) Satisfaction with body image (+) Age (NS) Weight (NS) BMI (NS) Easy Access to exercise location (NS)

[25] Brassington et al. 2002 U.S.		**Design: **Intervention study **Age: **70.2 (4.1) **Quality score: **38	**Intervention:** Endurance and strengthening exercise prescription or stretching and flexibility exercise prescription combined with telephone counselling to overcome exercise barriers for the promotion of exercise adherence **Outcome measure:** Self-report Exercise logs, validation through class-attendance and 'vitalogs' **Outcome:** Adherence to prescribed exercise (EX)	Change in exercise self-efficacy (+) Self-reported fitness outcome realizations (+) Baseline exercise self-efficacy (NS) Baseline fitness outcome expectancies (NS) Baseline psychological outcome expectancies (NS) Baseline social support for exercise (NS) Change in social support (NS)

[12] Burton et al. 1999 U.S.		**Design:**Observational study **Age: **[65 - 85^+^] **Quality score: **49	**Intervention:** Not applicable **Outcome measure:** Self-report 'how often do you perform physical activity such as walking briskly, gardening, or heavy housework?' **Outcome:** Performing brisk physical activity at least three times a week = active (less is considered sedentary or insufficiently active) (PA)	Age (-) Gender (male) (+) Marital status (+) General physical health (+) Belief in importance of physical activity (+) Emotional distress (-) Advice of physician about getting more exercise (-) Ethnicity (NS) Education (NS) Self-mastery (NS) Having a confidant (NS)

[38] Cheung et al. 2006 U.S.		**Design: **Randomized controlled trial **Age: **75.1 (7.0) **Quality score: **25	**Intervention:** Walking prescription and instruction with weekly prompting by nurse or computer **Outcome measure:** Self-report Exercise logs walking behaviour **Outcome:** Walking behaviour (miles, minutes and perceived exertion) and adherence to prescription (EX)	Exercise self-efficacy (+) Stage of change (NS)

[39] Costanzo and Walker 2008 U.S		**Design: **Randomized controlled trial **Age: **[50-65] **Quality score: **50	**Intervention:** Behavioural counselling to increase exercise self-efficacy and social support from friends and family **Outcome measure:** Self-report Modified 7-day activity recall **Outcome:** Self-reported moderate or greater physical activities of at least 4 MET reported in minutes based on Modified 7-day activity interview, listing several common activities: "moderate" (eg, cleaning, fishing, raking); "hard" activities (eg, golf, scrubbing); and "very hard" (eg, swimming) (PA/EX)	Change in exercise self-efficacy (+) Change in social support (family) (-) Change in social support (friends) (NS)

Cluster 1*	[42] Garcia and King 1991 U.S.	**Design: **Randomized controlled trial **Age: **56.4 (4.2) **Quality score: **41	**Intervention:** Personalized exercise prescription class-based or home-based (home-based included regular telephone contact) **Outcome measure:** Self-report Exercise logs validation through 'vitalogs' **Outcome:** Exercise performed relative to prescription (EX)	Marital status (+) Smoking status (-) Exercise self-efficacy (+) Exercise intervention condition (home-based vs class-based) (+) Age (NS) Gender (NS) Ethnicity (NS) Education (NS) Income (NS) BMI (NS) Self-motivation (NS) Exercise intervention condition (low vs high intensity) (NS) Perceived exertion, enjoyment and convenience of intervention (NS)
	
	[28] King et al. 1997 U.S.	**Design: **Intervention study **Age: **[50-65] **Quality score: **40	**Intervention:** Personalized exercise prescription class-based or home- based (home-based included regular telephone contact) **Outcome measure:** Self-report Exercise logs validation through 'vitalogs' **Outcome:** Exercise adherence to at least two thirds of exercise prescription (EX)	Exercise intervention condition (home-based vs class-based) (+) Stress (-) General physical functioning (+) Education (-) BMI (-) Age (NS) Gender (NS) Marital status (NS) Employment status (NS) Social support (NS)
	
	[45] Oka et al. 1995 U.S.	**Design: **Randomized controlled trial **Age: **[50-65] **Quality score: **21	**Intervention:** Personalized exercise prescription group-based or home- based (home-based included telephone counselling related to meeting exercise prescription) **Outcome measure:** Self-report Exercise logs validation through 'vitalogs' **Outcome:** Exercise adherence as percentage of exercise prescription (EX)	Smoking status (-) Exercise intervention condition (home-based vs class-based) (+) Preferring a lesser amount of social support from staff (+) Initial and continued social support (friends/family/staff) (+) Family satisfaction (-) Gender (NS)
	
	[32] Oman and King 1998 U.S.	**Design: **Intervention study **Age: **56.2 (4.2) **Quality score: **38	**Intervention:** Personalized exercise prescription class-based or home-based (home-based included telephone counselling related to meeting exercise prescription) **Outcome measure:** Self-report Exercise logs validation through 'vitalogs' **Outcome:** Exercise performed relative to prescription (EX)	Exercise self-efficacy (+) Exercise intervention condition (home-based vs class-based) (+) Prior exercise adherence (+) Exercise intervention intensity (NS) Exercise self-efficacy (+) Affect (NS)

Cluster 2*	[44] McAuley et al. 2003 U.S.	****Design: ****Randomized controlled trial **Age: **66.7 (5.4) **Quality score: **38	**Intervention:** Exercise classes three times a week either walking or a stretching and toning program **Outcome measure:** Self-report PASE (including leisure, household, and occupational activity) **Outcome:** Level of activity based on PASE (PA/EX)	Social support (NS) Prior exercise adherence (NS) Level of physical activity at 2 years after intervention (+)
	
	[30] McAuley et al. 2007 U.S.	**Design: **Intervention study **Age: **66.7 (5.4) **Quality score: **56	**Intervention:** Exercise classes three times a week either walking or a stretching and toning program **Outcome measure:** Self-report PASE (including leisure, household, and occupational activity) **Outcome:** Level of activity based on PASE (PA/EX)	Positive affect 2 years after intervention (+) Exercise self-efficacy 2 years after intervention (+) Exercise intervention condition (Walking vs Stretching) (+) Age (NS) Gender (NS) Ethnicity (NS) Marital status (NS) Education (NS) Income (NS)

[40] Dubbert et al. 2002 U.S.		**Design: **Randomized controlled trial **Age: **68.7 (4.7) **Quality score: **41	**Intervention:** Instruction video and individualized walking program with prompting phone calls or personalized phone calls with nurse counselling following stage of change **Outcome measure:** Self-report Walking diary, validation through peers and accelerometers with additional assessment using the 7-day Physical Activity Recall (PAR) **Outcome:** Episodes of at least 10 minutes duration walking for exercise (EX)	Walking companion (+) BMI (reduction) (+) Change in mobility (improvement) (+) Change in general physical health (improvement) (+) Exercise intervention condition (phone calls vs. no phone calls) (+) Smoking status (NS) Change in general mental health (NS)

[26] Emery et al. 1992 U.S.		**Design: **Intervention study **Age: **67.0 (4.9) **Quality score: **19	**Intervention:** Aerobics exercise group (3 supervised exercise sessions for 4 months), a yoga control or a waiting list control group **Outcome measure:** Self-report Retrospective self-report of physical activity during prior 12 months **Outcome:** Number of months, days per month, and minutes per day of physical activity (measure for overall activity and specific activities for which the participants were trained) converted to energy expenditure (PA/EX)	Cardiorespiratory endurance (+) Motor speed (+) Anxiety (-) Age (NS) Gender (NS) Prior exercise adherence (NS) General mental health (NS) General cognitive functioning (NS)

[41] Finkelstein et al. 2008 U.S.		**Design: **Randomized controlled trial **Age: **[50-85] **Quality score:** 63	**Intervention:** Financial incentive for minutes walking, jogging or running **Outcome measure:** Objective Pedometer (with paper back-up logs in case of technical problems) **Outcome:** Minutes logged walking, jogging or running (PA/EX)	Education (-) Income (-) Employment status (-) Exercise intervention condition (financial incentive vs. no-incentive) (+) Age (NS) Gender (NS)

[13] Hirvensalo et al. 2000 Finland		**Design: **Observational study **Age: **[65-84] **Quality score: **19	**Intervention:** Not applicable **Outcome measure:** Self-report The level of physical activity and its intensity was assessed using a six-point scale: 1) moving only in connection with necessary chores, 2) walking or other outdoor activities 1-2 times/week, 3) walking or other outdoors activities several times/week, 4) 1-2 times/week to the point of perspiring and heavy breathing, 5) several times/week to the point of perspiring and heavy breathing, 6) keep-fit exercise or competitive sport several times a week. **Outcome:** Level of physical activity based on categories of 6 point scale (PA/EX)	Age (-) Cardiovascular/musculoskeletal diseases (-) Competitive sports early in life (+) Recreations sports in adulthood (+)

[27] Jancey et al. 2007 Australia		**Design: **Intervention study **Age: **[65-74] **Quality score: **39	**Intervention:** Prescription walking intervention (aerobic, balance, strength and flexibility components), with trained walk leaders, offering advice, reassurance, encouragement, feedback and health education. Including non-monetary incentives and gatherings to enhance social support **Outcome measure:** Self-report IPAQ **Outcome:** Level of activity based on IPAQ (including occupational, household and leisure activity) (PA/EX)	Socioeconomic status (+) BMI (-) Loneliness (-) Walking self-efficacy (-) Baseline activity level (+) Age (NS) Gender (NS)

[14] Kahana et al. 2005 U.S.		**Design: **Observational study **Age: **79.1 (3.1) **Quality score: **38	**Intervention:** Not applicable **Outcome measure:** Self-report The total number of hours per week: walking, swimming, golfing, running/jogging, aerobics, stretching or calisthenics, weight lifting, dancing, biking, and other exercises. **Outcome:** The total number of hours per week (PA/EX)	Gender (male) (-) Future orientation (+) Age (NS) General physical health (NS)

[43] Li et al. 2001 U.S.		**Design **Randomized controlled trial **Age: **72.8 (5.1) **Quality score: **25	**Intervention:** Tai Chi practice sessions twice a week **Outcome measure:** Class-attendance recorded by instructor **Outcome:** Participants practise session attendance (EX)	Change in exercise self-efficacy (+)

[15] Li et al. 2005 U.S.		**Design: **Observational study **Age: **73.9 (2.6) **Quality score: **47	**Intervention:** Not applicable **Outcome measure:** Self-report Neighbourhood walking (walking or strolling though neighbourhood, walked or done physical activities with neighbours or gone to the park for walks or other physical activities) **Outcome:** Neighbourhood walking over the past 12 months (PA)	Neighbourhood safety for walking (+) Access to exercise facilities (+) Education (-) Neighbourhood social cohesion (NS) Exercise self-efficacy (NS) General physical health (NS) Income (NS)

[29] Lucidi et al. 2006 Italy		**Design: **Intervention study **Age: **[65-90] **Quality score: **50	**Intervention:** Sport activity program with two sessions per week including aerobic exercise, strength training, balance and flexibility **Outcome measure:** Self-report Class-attendance recorded by instructor **Outcome:** Percentage of attended sessions divided by number of possible sessions over the three months of exercise classes (EX)	Intention (+) Perceived behavioural control (NS) Exercise self-efficacy (NS) Exercise attitude (NS) Subjective norm (NS)

[16] Michael et al. 2010 U.S.		**Design: **Observational study **Age: **[65^+^] **Quality score: **75	**Intervention:** Not applicable **Outcome measure:** Self-report 2 questions of the PASE "Over the past 7 days, how often did you walk outside your home or yard for any reason. For example for fun or exercise, walking to work, walking the dog, etc.?" "On average, how many hours per day did you spend walking?" **Outcome: ** Walking behaviour (PA/EX)	Proximity of parks and trails (+) Proximity of recreational facilities (NS)

[31] Morey et al. 2003 U.S.		**Design: **Intervention study **Age: **71.5 (4.9) **Quality score: **32	**Intervention:** Exercise intervention to improve physical functioning supervised phase (followed by home-based phase for one of the two randomized groups) **Outcome measure:** Self-report validation through measured oxygen uptake **Outcome:** Following exercise prescription (EX). Participants were classified as adherent if their exercise averaged 20 minutes or more, 3 or more days a week, over six months	Gender (male) (+) Depression (-) BMI (-) General physical functioning (+) Chronic diseases (-) Pain (-) Prior exercise adherence (weekend exercise home-work) (+) Age (NS) Ethnicity (NS) General physical health (NS) Exercise self-efficacy (NS) Locus of Control (NS) Social support (NS) Exercise intervention condition (aerobic only vs aerobic + flexibility) (NS)

[17] Nitz and Choy 2007 Australia		**Design: **Observational study **Age:** [40-80] **Quality score: **15	**Intervention:** Not applicable **Outcome measure:** Self-report The level of physical activity and its intensity was assessed using a six-point scale: 1) moving only in connection with necessary chores, 2) walking or other outdoor activities 1-2 times/week, 3) walking or other outdoors activities several times/week, 4) 1-2 times/week to the point of perspiring and heavy breathing, 5) several times/week to the point of perspiring and heavy breathing, 6) keep-fit exercise or competitive sport several times a week. **Outcome:** Level of physical activity based on categories of 6 point scale (PA/EX)	BMI (-) Baseline activity level (+) Change in BMI (NS) Age (NS) Number of falls (NS) Number of falls since baseline (NS) Stability (NS) Number of medical conditions (NS) Increase in number of medical conditions (NS) Number of medications (NS) Change in number of medications (NS)

[33] Oman and King 2000 U.S.		**Design: **Intervention study **Age: **65.5 (4.3) **Quality score: **25	**Intervention:** Personalized exercise prescription class-based or home-based (home-based included telephone counselling related to meeting exercise prescription) **Outcome measure:** Exercise logs validation through 'vitalogs' **Outcome:** Exercise performed relative to prescription (EX)	Major life events (-)

[34] Rhodes et al. 2001 Canada		**Design: **Intervention study **Age: **76.4 (1.6) **Quality score: **25	**Intervention:** Three weekly sessions of progressive resistance training **Outcome measure:** Class-attendance **Outcome:** Adherence measured through attendance (EX)	Prior exercise adherence (+) Baseline exercise self-efficacy (+) Initial general social support (+) Continued general and program social support (NS)

[35] Sarkisian et al. 2007 U.S.		**Design: **Intervention study **Age: **[65^+^] **Quality score: **50	**Intervention:** Attribution retraining followed by physical activity class including strength, endurance and flexibility **Outcome measure:** Objective Pedometer **Outcome:** Weekly step count (PA)	Positive age expectations (+)

[18] Shaw and Spokane 2008 U.S.		**Design: **Observational study **Age: **[54-72] **Quality score: **48	**Intervention:** Not applicable **Outcome measure:** Self-report Single item, vigorous physical activity or exercise, 3 times a week or more, over the past 12 months (PA/EX) **Outcome:** Vigorous physical activity or exercise 3 times a week or more, over the past 12 months (yes or no) (PA/EX)	Age (-) Education (+) Employment status (+) Change in employment status (+) Chronic conditions (-) Change in chronic conditions (increase) (-) General physical functioning (+) General physical functioning (increase in limitations)(+) Depressive symptoms (-) Change in depressive symptoms (increase) (-)

[19] Shimada et al. 2007 Japan		**Design: **Observational study **Age: **[70^+^] **Quality score: **54	**Intervention:** Not applicable **Outcome measure:** Self-report Regular physical activity (yes/no, frequency and nature of activity: golf, ball games, hiking, home-based or group exercise, dancing, swimming, martial arts, jogging, walking, other exercise) **Outcome:** Regular physical activity was defined as carrying out any type of physical activity 5 times or more per week (PA/EX)	Gender (male) (+) Smoking status (-) Physical functioning (slow walking speed) (-) Fear of falling (-) Age (NS) General physical health (NS)

[20] Stiggelbout et al. 2006 The Netherlands		**Design: **Observational study **Age: **60.9 (8.4) **Quality score: **50	**Intervention:** Not applicable **Outcome measure:** Self-report Part of the Dutch Monitor on Physical Activity and Health to assess compliance with Dutch public-health guidelines **Outcome:** Norm-active is defined as performing 30 minutes or more of moderate-intensity physical activity on most, and preferably all, days - either in a single session or accumulated in multiple bouts of al least 10 minutes (PA/EX)	Prior exercise adherence (+) Perceived quality of the program (+) Exercise attitude (+) Exercise barriers (-) Exercise intention (+) Exercise self-efficacy (NS) Coping (NS) Social influences and support (NS)

[21] Touvier et al. 2010 France		**Design: **Observational study **Age:**[45-64] **Quality score:**46	**Intervention:** Not applicable **Outcome measure:** Self-report MAQ Past 12 month physical activity during leisure time and at work **Outcome:** Subjects were considered to meet overall PA recommendations if their overall PA was ≥ 60 min per week of vigorous activities with at least 20 min per session or ≥ 150 min per week of moderate activities (PA/EX)	Retirement (+) Gender (NS) Occupation physical demand level (NS)

[22] Tu et al. 2004 U.S.		**Design: **Observational study **Age: **63.7 (7.3) **Quality score: **52	**Intervention:** Not applicable **Outcome measure:** Class-attendance recorded by research assistant **Outcome:** Measured attendance (EX)	Weather conditions (good) (+) High blood pressure (-) Age (NS) Ethnicity (NS) General physical health (NS) Perceived barriers (NS) Pain as exercise barrier (NS) Exercise self-efficacy (NS) Workers going to work on foot in neighbourhood (NS)

[36] Wilcox and King 2004 U.S.		**Design: **Intervention study **Age: **70.2 (4.1) **Quality score: **25	**Intervention:** Exercise classes aerobic and strength training or stretching and relaxation combined with home-work with telephone counselling to encourage participation in the program **Outcome measure:** Self-report Exercise logs validation through class-attendance and 'vitalogs' **Outcome:** Participation calculated as percentage of exercise sessions completed divided by sessions prescribed (EX)	Number of life events (-) Interpersonal loss (-)

[37] Williams and Lord 1995 Australia		**Design: **Intervention study **Age: **71.6 (5.5) **Quality score: **16	**Intervention:** Exercise to improve balance, coordination, strength and cardiorespiratory fitness **Outcome measure:** Class-attendance and activities outside the program **Outcome:** Adherence was defined as number of exercise classes attended (EX)	Age (-) Reaction time (+) Psychoactive drug use (-) Physical strength (+) Cognitive reasoning ability (+) Depression (-) Self reported general physical health outcome realizations (+) Self reported physical functioning outcome realizations (+) Self reported cognitive functioning outcome realizations (+) Self reported psychological outcome realizations (+) Education (NS) BMI (NS) General physical health (NS) General mental health (NS) General cognitive functioning (NS) Baseline activity level (NS) Locus of control (NS)

[23] Yasunaga et al. 2008 Japan		**Design: **Observational study **Age: **[65-83] **Quality score: **88	**Intervention:** Not applicable **Outcome measure:** Objective Pedometer/accelerometer **Outcome:** Number of steps taken and the intensity of physical activity (PA)	Age (-) Gender (male) (+) Weather conditions (good) (+)

* Cluster of multiple manuscripts reporting on the same original data set

**Table 3:** Methodological quality of the included studies

		Item*								Quality score %
**Reference**		**1**	**2**	**3**	**4**	**5**	**6**	**7**	**8**	

Boyette et al., 1997		?	+	+	-	0.17	0.17	-	-	29

Brassington et al., 2002		?	+	?	-	0	0	+	+	38

Burton et al., 1999		?	-	+	-	0.45	0.45	+	+	49

Cheung et al., 2007		?	+	?	-	0	0	+	-	25

Costanzo and Walker 2008		+	+	?	-	0	0	+	+	50

Cluster 1**	Garcia and King, 1991	?	?	?	-	0.69	0.62	+	+	41

	King et al., 1997	?	?	?	-	0.6	0.6	+	+	40

	Oka et al., 1995	-	?	?	-	0.33	0.33	+	-	21

	Oman and King, 1998	-	?	?	-	0.5	0.5	+	+	38

Cluster 2**	McAuley et al., 2003	?	+	?	-	0	0	+	+	38

	McAuley et al., 2007	?	+	?	-	0.7	0.8	+	+	56

Dubbert et al., 2002		?	+	?	-	0.14	0.14	+	+	41

Emery et al., 1992		?	+	?	-	0.25	0.25	-	-	19

Finkelstein et al., 2008		?	+	?	+	1	1	+	-	63

Hirvensalo et al., 2000		?	?	?	-	0.25	0.25	+	?	19

Jancey et al., 2007		-	-	-	-	0.71	0.43	+	+	39

Kahana et al., 2005		?	?	?	-	0.50	0.50	+	+	38

Li et al., 2001		?	-	-	-	0	0	+	+	25

Li et al., 2005		?	+	?	-	0.43	0.29	+	+	47

Lucidi et al., 2006		+	+	?	-	0	0	+	+	50

Michael et al., 2010^§^		?	+	+	-	1	1	+	+	75

Morey et al., 2003		-	+	?	-	0.29	0.29	+	-	32

Nitz and Choy, 2007		?	+	?	-	0.09	0.09	-	-	15

Oman and King, 2000		?	+	?	-	0	0	+	-	25

Rhodes et al., 2001		?	+	?	-	0	0	+	-	25

Sarkisian et al., 2007		?	+	?	+	0	0	+	+	50

Shaw and Spokane, 2008		+	?	?	-	0.40	0.40	+	+	48

Shimada et al., 2007		+	-	?	-	0.67	0.67	+	+	54

Stiggelbout et al., 2006		+	+	?	-	0	0	+	+	50

Touvier et al., 2010		?	?	?	-	0.67	1	+	+	46

Tu et al., 2004		?	?	?	-	0.56	0.56	+	+	39

Wilcox and King, 2004		?	+	?	-	0	0	+	-	25

Williams and Lord, 1995		?	-	?	-	0.12	0.12	+	-	16

Yasunaga et al., 2008^§^		?	+	+	+	1	1	+	+	88

* Item number corresponds with item description in Table 1

** Cluster of multiple manuscripts reporting on the same original data set

**§ **Manuscripts with a percentage of 70% or more were considered high quality

## Results

The initial search retrieved 8278 manuscripts (see flowchart in Figure 1). Thirty-four manuscripts reporting on 30 studies, met the inclusion criteria (19 manuscripts obtained from PubMed/Medline, 13 manuscripts from PsycINFO/OVID and 2 manuscripts through the reference lists of reviews retrieved in the search). Of the included manuscripts, 12 were observational [12-23], 14 were intervention [24-37] and 8 were RCTs [38-45]. Table 2 presents the characteristics of the included manuscripts.

### Outcome measure

PA was reported in four manuscripts [12,15,23,35]. EX was reported in 16 manuscripts [22,24,25,28,29,31-34,36-38,40,42,43,45] and a combination of PA and EX (PA/EX) was reported in 14 manuscripts [13,14,16-21,26,27,30,39,41,44].

Three out of the 34 manuscripts objectively measured PA or PA/EX using pedometers or accelerometers [23,35,41]. Twenty-two manuscripts used self-report measures, such as questionnaires, exercise logs or class attendance logs completed by the participants [12-22,24,26,27,29,30,34,37-39,43,44]. In nine manuscripts a self-report measure was combined with validation through class attendance recorded by the instructor and/or data derived from a pedometer/accelerometer/oxygen uptake ([25,28,31-33,36,40,42,45]; see Table 2).

### Methodological quality assessment

The methodological quality scores are presented in Table 3. Initial disagreement (25%) between the raters was based on differences in reading and interpretation. The majority of the initial disagreement was based on items 5 and 6 (were the determinants of PA or EX measured with a reliable and valid tool). The methodological quality score ranged from 15 to 88%. Two studies received scores above 70%; a score considered to indicate "high" quality [16,23].

### Determinants of PA

Supported by one high quality study [23] and one low quality study [12], moderate evidence was found for a positive association between male *gender *and PA, as well as younger *age *and PA. For all other possible determinants of PA we found insufficient evidence.

### Determinants of EX

Based on four low quality studies [22,24,28,31,42] (two manuscripts reported on the same study), we found no evidence for an association between *age *and EX. Based on three low quality studies we found no evidence for a relation between *ethnicity *and EX [22,31,42].

Based on three low quality studies, we found no relation between *general physical health *and EX [22,31,37]. Four low quality studies provided moderate evidence for a positive association between *general physical functioning *and EX [28,31]. Moderate evidence for a negative association between *(chronic) conditions and diseases *and EX was observed [22,31]. Based on two low quality studies moderate evidence was found for a negative association between *depression *and EX [31,37]. We found moderate evidence for a positive association of *prior exercise adherence *[31,32] and *self-reported beneficial health or physical functioning outcomes *[25,37] with EX. The four studies supporting this evidence were of low methodological quality.

Moderate evidence, based on two low quality studies, was found for a positive association between *change in exercise self-efficacy *and EX [25,43]. Based on two low quality studies we found no evidence for an association between *locus of control *and EX [31,37]. *Major life events *were reported in two low quality studies [33,36]. Based on these studies, moderate evidence was found for a negative association between major life events and EX. For all other determinants assessed, insufficient evidence was found.

### Determinants of PA/EX

Based on seven low quality studies [14,17,19,26,27,41], we found no evidence for an association between *age *and PA/EX. Based on two low quality studies, we found moderate evidence for a negative association between *BMI *and PA/EX [17,27]. Supported by two low quality studies we found no evidence for an association between *general physical health *and PA/EX [14,19]. We found moderate evidence for a positive association between *baseline activity *(based on two low quality studies; [17,27] and PA/EX.

We found no evidence for an association between *baseline social support *and PA/EX, based on two low quality studies [20,44]. For all other possible determinants of PA/EX we found insufficient evidence.

## Discussion

The aim of this review was to assess the determinants of physical activity (PA) as well as exercise (EX) in healthy older adults, considering the methodological quality of the included studies. The 34 manuscripts included in this review provide an overview of the determinants of PA or EX that have been studied in the last 20 years. Although we set out to differentiate between determinants of PA and EX, we concluded "insufficient evidence" for most associations between possible determinants and PA or EX. This was mainly due to multiple low quality studies reporting inconsistent findings, lack of high quality studies, and often only one manuscript reporting on a particular determinant. The limited evidence available on PA and EX revealed a dissimilarity concerning the determinant age; moderate evidence was found for a positive association with younger age and PA, whereas no evidence was found for an association of age and EX. No inverted associations were found between determinants of PA and EX. No further illustration could be made of similarities or dissimilarities between PA and EX. This emphasises the need for additional research, particularly on determinants of PA.

We have categorised PA and EX according to the definitions by Caspersen et al. [8]. These definitions have been adopted by the ACSM [3]. In addition to the possible differences in (determinants of) PA versus EX, differences within PA and EX categories may exist. Indeed, we have included four manuscripts reporting on PA in which different behavioural outcomes were assessed (step count, walking, gardening, heavy housework, neighbourhood walking and engaging in activities with neighbours). As of yet, there is too little high-quality research to differentiate within activity categories. In future research, more differentiation is desirable. Not withstanding these methodological issues, the renewed focus on PA (as opposed to EX) could be important since recent work on the (cost-) effectiveness of PA and EX interventions showed the long term beneficial effect of PA over EX. In many behavioural-based lifestyle interventions, participants are taught to integrate PA in their daily lives [46-48]. Especially activities such as walking, gardening or housework could be very well integrated in the lives of older adults.

Overall, the methodological quality of the included studies was low, with only two manuscripts scoring above 70% (high quality). Determinants were often measured by different instruments, complicating comparison between studies. Moreover, most of the determinants were assessed using instruments with unknown or poor reliability and validity. The most commonly used instruments assessing PA or EX behaviour were self-report measures. Unfortunately, there are currently no self-report instruments to assess PA or EX in older adults that are both reliable and valid [49]. Instruments like pedometers and accelerometers, which generally perform better in terms of reliability and validity [50-53], are therefore increasingly used to assess PA or EX [54,55]. Applying a strict methodological quality assessment could have led to overly cautious conclusions on the evidence in the field. Although the quality assessment is one of many ways to judge the strength of evidence, and has its own methodological restrictions, we do feel that future research on determinants of PA and EX in older adults could be greatly improved.

We recommend additional research on the determinants of PA. We further recommend the development and use of valid and reliable measurement instruments for determinants as well as the use of objective measurement instruments of PA and EX. The use of measurement instruments with good psychometric properties and the use of comparable, and preferably similar, instruments assessing determinants should allow for a better comparison between studies.

Future interventions should carefully build upon the limited evidence available. Some of the assessed determinants (e.g. gender and age) suggest the targeting of subgroups within the population for interventions. The Intervention Mapping protocol [56] provides guidelines and tools for theory based development of programme materials and activities to increase physical activity and exercise. This approach can be used to systematically explain and change modifiable determinants of PA or EX (such as exercise self-efficacy).

In our review we have identified many determinants of PA and EX in healthy older adults (demographic determinants; determinants of health and health behaviour; psychological determinants; social determinants; environmental determinants; determinants related to the intervention). However, there is evidence available on possible other determinants of physical activity and exercise behaviour that we could not identify in our search. These include genetic determinants [57-59], policy related determinants [60,61] and, in addition to determinants of planned behaviour change[62], unplanned or unintentional behavioural change [63-67]. Recent work has shown the possibilities of trans-disciplinary research integrating theoretical approaches (e.g. genetic, physiological, psychological) in physical activity research that could provide a framework for further research [68,69].

Limitations inherent to writing a review consist of "publication bias" and the possibility that manuscripts may have been overlooked. We have tried to prevent the latter by selecting a medical and a psychological database, using broad search terms and checking reference lists from other reviews. Another limitation, specific to this review, is the selection of literature describing studies with a population aged 55 and older. Looking at this relatively broad age range combined, may have masked possible differences in determinants between subsamples of this age range. Additionally, excluding specific subsamples of the older population (i.e. confined geographic area or diagnosed diseases) may have further masked possible differences in determinants between subsamples.

## Conclusions

A large number of determinants of PA and EX were examined and for most determinants there was insufficient evidence. Assessed determinants of older adults' PA, EX and PA/EX reported in this review, such as age, BMI, exercise self-efficacy and social support, are similar to determinants reported by Trost et al. [70] for the entire adult population. Furthermore, they also resemble the determinants of initiation and maintenance of physical activity among older adults reported by Van Stralen et al. [7]. Unlike these earlier reviews we took the methodological quality of the manuscripts into account. This resulted in more cautious conclusions on the available evidence in the field.

Although a diverse set of possible determinants occurs in the literature (e.g., characteristics of the individual, of the social and physical environment and of the intervention), other possible determinants remain largely unstudied. There is a relative shortage of manuscripts assessing determinants of PA, which needs to be addressed in future research, ideally using objective, valid and reliable measures. Subsequently, the possible differences in determinants between PA and EX need further study as well.

## Competing interests

The authors declare that they have no competing interests.

## Authors' contributions

MK and MV conducted the literature search. All authors evaluated the appropriateness of included manuscripts. All authors contributed equally to the editing and approving of the final version of the paper.

## Authors' information

*Margot Koeneman*, *MSc*, is a PhD student at Body@Work research center, a joint initiative of TNO, Leiden, The Netherlands and VU University, and VU University Medical Centre, Amsterdam, The Netherlands. Her background is in Social Psychology. She previously worked as a junior researcher at the AMC medical centre, Amsterdam.

*Dr Marieke Verheijden *is working at TNO (Netherlands organisation for applied scientific research) in Leiden. Her professional background is in Nutrition and Health. Most of her work was on the development, implementation and evaluation of (tailored) health promotion programs, predominantly in the areas of prevention of overweight by promoting healthy nutrition and physical activity. She is also a staff member of the Body@Work research centre Physical activity, Work and Health TNO VU medical centre.

*Dr Mai Chin A Paw *is associate professor at the Department of Public and Occupational Health, EMGO Institute - VU University Medical Centre in Amsterdam, The Netherlands. Her background is in Human Movement Science and Epidemiology. She obtained her PhD in 1999 for her thesis that investigated the effects of physical exercise and micronutrient supplementation on the health of frail older people. Currently, she chairs the section Youth and Health within the department of Public and Occupational health and is involved in several research projects. Examples are the development and evaluation of strategies promoting physical activity and reducing sedentary behaviours, measurement of physical activity, and prevention and treatment of obesity. She is associate editor of The Journal of Science and Medicine in Sport and member of the editorial board of the International Journal of Behavioral Nutrition and Physical Activity.

*Professor Dr Marijke Hopman-Rock *is working at TNO (Netherlands organisation for applied scientific research) in Leiden and professor in Physical activity and health of older persons at the VU university medical centre in Amsterdam. She is also in the management team of the Body@Work research centre Physical activity, Work and Health TNO VU medical centre. Her professional background is in Biology, Psychology (statistics) and Epidemiology (PhD). As a part-time professor in Amsterdam she supervises several PhD students. She is also past (founding) chair of the European Network for Action on Ageing and Physical Activity (EUNAAPA) and associate partner in several EU projects in the area of physical activity. She is associate editor of the Journal of Aging and Physical Activity (JAPA) and BMC Public Health and is also on the board of other journals, including the Human Kinetics journal Active Aging Today. She is a member of the TOP ZonMw/NWO committee. In the past she was Head of the Department of PA and Health at TNO and program manager in the same area; Vice-president of the EGREPA (European group for Research in Elderly and PA) and co-chair of the Aging interest group of the American College of Sport and Exercise Medicine (ACSM).
